# The comparison of cytotoxicity of the anticancer drugs doxorubicin and ellipticine to human neuroblastoma cells

**DOI:** 10.2478/v10102-010-0036-9

**Published:** 2010-11

**Authors:** Jitka Poljaková, Tomáš Eckschlager, Jana Hřebačková, Jan Hraběta, Marie Stiborová

**Affiliations:** 1 Department of Biochemistry, Faculty of Science, Charles University, Prague, Albertov 2030, 128 40 Prague 2, CZECH REPUBLIC; 2 Department of Pediatric Hematology and Oncology, 2^nd^ Medical School, Charles University and University Hospital Motol, Prague 5, CZECH REPUBLIC

**Keywords:** anticancer drug, ellipticine, doxorubicin, human neuroblastoma cells, cytotoxicity

## Abstract

Ellipticine is an antineoplastic agent, whose mode of action is based mainly on DNA intercalation, inhibition of topoisomerase II and formation of covalent DNA adducts mediated by cytochromes P450 and peroxidases. Here, the cytotoxicity of ellipticine to human neuroblastoma derived cell lines IMR-32 and UKF-NB-4 was investigated. Treatment of neuroblastoma cells with ellipticine was compared with that of these cancer cells with doxorubicin. The toxicity of ellipticine was essentially the same as that of doxorubicin to UKF-NB-4 cells, but doxorubicin is much more effective to inhibit the growth of the IMR-32 cell line than ellipticine. Hypoxic conditions used for the cell cultivation resulted in a decrease in ellipticine and/or doxorubicin toxicity to IMR-32 and UKF-NB-4 neuroblastoma cells.

## Introduction

Neuroblastoma is the most frequent solid extra cranial tumor in children and is a major cause of death from neoplasia in infancy (Maris and Mathay, 1999). These tumors are biologically heterogeneous, with cell populations differing in their genetic programs, maturation stage and malignant potential (Brodeur, 2003). On the one hand, low risk neuroblastoma is one of the rare human malignancies known to demonstrate spontaneous regression from an undifferentiated state to a completely benign cellular formation (ganglioneuromas). On the other hand, high risk neuroblastoma grows relentlessly and may be rapidly fatal (Brodeur, 2003). Prognosis of high risk tumors is poor, because drug resistance arises in the majority of those patients, initially responding to chemotherapy, in spite of the intensive therapy including megatherapy with subsequent hematopoietic progenitor cell transplantation, biotherapy and immunotherapy (Brodeur, 2003).

Chemotherapy agents used in combination have been found to be effective against neuroblastoma. Agents commonly used are platinum compounds (carboplatin), alkylating agents (cyclophosphamide, ifosfamide, melphalan), topoisomerase II inhibitors (etoposide), anthracycline antibiotics (doxorubicin) and vinca alkaloids (vincristine). Some novel regimen include also topoisomerase I inhibitors (topotecan and irinotecan), which have been found to be effective against recurrent disease (Brodeur, 2003).

Ellipticine (5,11-dimethyl-6H-pyrido[4,3-b]carbazole) and several of its more soluble derivatives exhibit significant antitumor and anti-HIV activities (Stiborová *et al*., 2001). The main reason for the interest in ellipticine and its derivatives for clinical purposes is their high efficiencies against several types of cancer (Auclair, 1987). Nevertheless, ellipticine is a potent mutagen (for an overview, see Stiborová *et al*., 2001). Ellipticine is an agent which cause DNA damage by several modes of action. Intercalation and/or inhibition of topoisomerase II was thought to be the major mechanism of ellipticine action. These mechanisms of action do not however, explain the ellipticine selectivity to several cancer diseases. Recently we have showed that ellipticine also covalently binds to DNA after being enzymatically activated (Stiborová *et al*., 2001; Frei *et al*., 2002; Stiborová *et al*., 2003a; Stiborová *et al*., 2003b). Human cytochrome P450 (CYP) enzymes, CYP3A4, 1A1, and 1B1–enzymes, which are expressed in tumors sensitive to ellipticine (*i.e*., breast cancer; Murray *et al*., 1993), were found to be the most efficient CYPs activating ellipticine to form covalent DNA adducts (Stiborová *et al*., 2001). The formation of these adducts was also detected in V79 lung fibroblast cells transfected with human CYP3A4, 1A1, and 1A2 (Frei *et al*., 2002), in human breast adenocarcinoma MCF-7 cells (Borek-Dohalská *et al*., 2004), and *in vivo* in rats exposed to ellipticine (Stiborová *et al*., 2003b). On the basis of these data, ellipticine might be considered a drug whose pharmacological efficiencies and/or genotoxic side effects are dependent on its enzymatic activation in target tissues (Stiborová *et al*., 2001; Frei *et al*., 2002; Stiborová *et al*., 2003a; Stiborová *et al*., 2003b; Borek-Dohalská *et al*., 2004).

Some improvement in therapeutic options of neuroblasoma has been made in the last decade, but there is still the need for the development of new therapies. Therefore, the present study was undertaken to investigate the cytotoxicity of ellipticine on human neuroblastoma cell lines and to compare the cytotoxicity of ellipticine with that of doxorubicin to these cancer cells. Another aim of the study was to evaluate the effect of hypoxia on cytotoxicity produce by both agents.

## Material and methods

### Chemicals

Ellipticine was obtained from Sigma (St. Louis, MO, USA). Doxorubicin was obtained from EBEWE Pharma Ges.m.b.H. (Uunterach, Austria). All other chemicals used in the experiments were of analytical purity or better.

### Cell cultures

The UKF-NB-4 neuroblastoma cell line, established from bone marrow metastases of high risk neuroblastoma, was a gift of prof. J. Cinatl, Jr. (J. W. Goethe University, Frankfurt, Germany). Cell line UKF-NB-4 was established from infiltrated bone marrow of chemoresistant high risk neuroblastoma recurrency. IMR-32, high risk neuroblastoma derived cell line, was of the commercial source (LGC Promochem, Wesel, Germany). Both used cell lines were derived from a high risk neuroblastoma with MYCN amplification. The doxorubicin and/or ellipticine resistant cell sublines designated IMR-32 (DOXO), UKF-NB-4 (DOXO) and UKF-NB-4 (Elli) were established by incubation of parental cells with increasing concentrations of respective drug by the procedure as described (Kotchetkov *et al*., 2003). Cells were grown at 37°C and 5% CO_2_ in Iscove's modified Dulbecco's medium (IMDM) (KlinLab Ltd, Prague, Czech Republic), supplemented with 10% fetal calf serum, 2mM L-glutamine, 100 units/ml of penicilline and 100µg/ml streptomycine (PAA Laboratories, Pasching, Austria). For hypoxia experiments, cells were maintained in modular incubator chamber (Billups-Rothenberg Inc., Del Mar, CA, USA) flushed with 1% O_2_, 5% CO_2_ and balance N_2_ for 4 min (hereafter refered to as hypoxia). This chamber was placed into 37°C.

### MTT assay

The cytotoxicity of ellipticine and doxorubicin was determined by MTT test. For a dose-response curve, DMSO stock solutions of ellipticine (10mM) and aqueous solutions of doxorubicin (1 mM) were dissolve in culture medium to final concentrations of 0–50µM were added. Cells in exponential growth were seeded at 1×10^4^ per well in a 96-well plate. Briefly, after incubation (96 hours) at 37°C in 5% CO_2_ saturated atmosphere the MTT solution (2 mg/ml PBS) was added, the plates were incubated for 4 hours and cells lysed in 50% N,N-dimethylformamide containing 20% of SDS pH4.5. The absorbance at 570nm was measured for each well by multiwell ELISA reader Versamax (Molecular devices, CA). The mean absorbance of medium controls was the background and was subtracted. The absorbance of control cells was taken as 100% viability and the values of treated cells were calculated as a percentage of control. Each value is the mean of 8 wells with standard deviation. The IC_50_ values were calculated from the linear regression of the dose-log response curves by SOFTmaxPro.

### Flow cytometry

Cells were harvested by trypsinization and washed with PBS. FITC labeled antibody against ABCB1 (anti P-gp FITC, Immunotech) was added and samples were incubated for 15 minutes at room temperature. After washing with PBS the cells were resuspended in PBS and analyzed by flow cytometry FACSCalibur (BD).

## Results

### Cytotoxicity of ellipticine to human neuroblastoma cells

**Figure 1:**
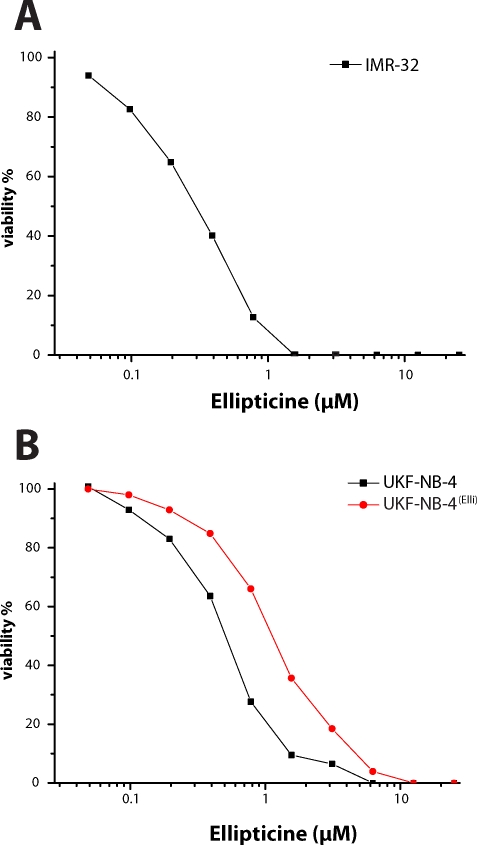
Cytotoxicity (viable cells as percentage of control) of ellipticine to IMR-32 (A), UKF-NB-4 and UKF-NB-4 cells resistant to ellipticine (UKF-NB-4^Elli^) (B) after 96 h exposure to the compound, determined by the MTT assay. Values are means of 8 determinations. Standard deviation was ≤ 10%.

To determine the cytotoxicity of ellipticine to human neuroblastoma cells, these cells were treated with increasing concentrations of ellipticine. We first determined the effect of ellipticine on growth of human neuroblastoma cell lines (IMR-32 and UKF-NB-4) cultured for 96 h in the presence of ellipticine, using MTT assay. As shown in Figure 1, both neuroblastoma cell lines were sensitive to ellipticine. Cytotoxicity of ellipticine was compared with that of doxorubicin, one of the drugs currently used for neuroblastoma treatment (Table 1). Besides the effects of ellipticine or doxorubicin to these parental neuroblastoma cell lines, derived daughter neuroblastoma lines that were resistant to doxorubicin were also investigated. Ellipticine and doxorubicin inhibited the growth of neuroblastoma cell lines in a dose-dependent manner. The IC_50_ values for ellipticine and doxorubicin calculated from the dose-log response curves are shown in Table 1. The toxicity of ellipticine to UKF-NB-4 cells was analogous to that of doxorubicin to these cell lines, while IMR-32 cells were much more sensitive to the treatment with doxorubicin than with ellipticine (see IC_50_ values shown in Table 1).

**Table 1:** Cytotoxicity of ellipticine and doxorubicin to neuroblastoma cell lines.

Cells	IC_50_(µM)	
	
	for ellipticine	for doxorubicin
IMR-32	0.27 ± 0.02	0.02 ± 0,01
IMR-32(hypoxic conditions)	0.43 ± 0.02	not measured
IMR-32 (DOXO)	0.53 ± 0.03	0.03 ± 0.01
UKF-NB-4	0.44 ± 0.03	0.70 ± 0.07
UKF-NB-4(hypoxic conditions)	0.77 ± 0.03	1.61 ± 0.08
UKF-NB-4 (DOXO)	1.14 ± 0.07	3.80 ± 0.38
UKF-NB-4 (Elli)	1.17 ± 0.07	0.70 ± 0.07
UKF-NB-4 (Elli)(hypoxic conditions)	1.57 ± 0.14	1.51 ± 0.08

IC_50_ values were calculated from the linear regression of the dose-log response curves after 96 h exposure to the compound, determined by the MTT assay. Values are mean ± S.D. of at least 3 experiments.

A decrease in the viability induced by ellipticine and/or doxorubicin in the parental cell lines was compared with that in their variants resistant to doxorubicin. Interestingly, a decrease in sensitivity of the doxorubicin-resistant lines to ellipticine was significantly lower than a decrease in sensitivity of these lines to doxorubicin (Table 1), even though the cross-resistance would be expectable (Kotchetkov *et al*., 2003). In the case of the UKF-NB-4 cell line, the IC_50_ value for ellipticine in UKF-NB-4 (DOXO) was only 2.6-fold higher than that in the parental UKF-NB-4 cell line, whereas the IC_50_ value of doxorubicin increased by 5.4-fold in the doxorubicin resistant cell line.

### Ellipticine induces resistance to this compound in neuroblastoma cells

**Figure 2:**
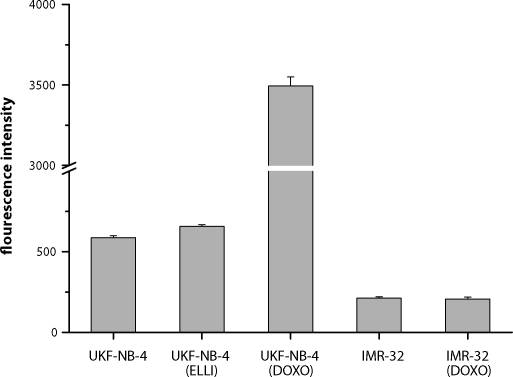
Levels of P-glycoprotein expressed as mean fluorescence intensity in neuroblastoma derived cell lines.

In order to evaluate the potential of ellipticine to induce the resistance of neuroblastoma cells to this drug, one of the cell lines, UKF-NB-4, was incubated 36 months with increasing concentrations of ellipticine (1–2.5µM). A 2.6-fold increase in the IC_50_ value for ellipticine was found (Table 1, Figure 1). The induction of resistance of the UKF-NB-4 cell line to ellipticine is not mediated by P-glycoprotein, a member of ABC transporters (Figure 2).

### Ellipticine resistance of UKF-NB-4 is not mediated by P-glycoprotein

Lower cytotoxicity of doxorubicin to UKF-NB-4 (DOXO) than to UKF-NB-4 is caused by higher expression of P-glycoprotein in a membrane of the doxorubicin resistant cell line. In contrast to these results, the IMR-32 (DOXO) cell line expresses P-glycoprotein at the same level as parental IMR-32 line. The UKF-NB-4 cell line resistant to ellipticine also does not express P-glycoprotein in the higher levels than the parental cell line. Resistance to ellipticine is therefore mediated by another mechanism of action. The study resolving this mechanism is under the way in our laboratories.

### Hypoxia decreases the toxicity of ellipticine and doxorubicin

Hypoxia was found in most tumor malignancies. To determine the effect of hypoxia on the cytotoxicity of ellipticine and/or doxorubicin to IMR-32, UKF-NB-4 and daughter cells resistant to doxorubicin, the cells were treated with increasing concentrations of either cytostatics for 96 h under hypoxic conditions and analyzed using MTT assay. As shown in Table 1, all neuroblastoma cell lines were less sensitive to applied cytostatics under the hypoxic conditions of their cultivation.

## Discussion

The results of this study show that ellipticine is cytotoxic to human neuroblastoma cell lines (both to the parental IMR-32 and UKF-NB-4 lines and their variants resistant to doxorubicin), inhibiting the growth of these cancer cells.

Hypoxia frequently occurs in tumors because of their fast growth and inadequate vascularisation. It strongly correlates with advanced disease and poor outcome caused by chemoresistance. The cytotoxicity of ellipticine or doxorubicin decreased when neuroblastoma cells were cultivated and treated with these agents under the limited concentration of oxygen.

Because drug resistance is general feature of neuroblastoma, and arises in the majority of patients suffering from this cancer, we also investigated whether ellipticine is capable of inducing resistance in neuroblastoma cells. The UKF-NB-4 cell line was used for such a study. The results demonstrate that ellipticine might, to some extent, induce the drug resistance in these cells. Long-term treating this cancer line with increasing concentrations of ellipticine resulted in the morphological changes in the ellipticine-resistant cell line (not shown). The decrease in sensitivity of this cell line to ellipticine was, however, lower than that in sensitivity of the doxorubicin-resistant UKF-NB-4 cell line to doxorubicin. The doxorubicin-induced resistance of these neuroblastoma cells resulted in more than 5-times higher decrease in sensitivity of neuroblastoma to this drug. Moreover, the IC_50_ values for ellipticine in the ellipticine-resistant neuroblastoma cell line is, even under the hypoxic conditions, still one order of magnitude lower than the ellipticine levels in blood in mice, 4 hours after their p.o. treatment with ellipticine in a tolerable dose (Hardesty *et al*., 1972). The study resolving the reasons of the changes found in the ellipticine-resistant neuroblastoma cells on the molecular levels is under way in our laboratories. The changes in the genetic programs in this cell line after induction of resistance to ellipticine are analyzed at the present time. Our experiments suggest that the ellipticine-mediated resistance is not dependent on expression of P-glycoprotein as it is the case of the UKF-NB-4 cell line resistant to doxorubicin (Bedrníček *et al*., 2005, present paper).

The results presented in this paper are the first report demonstrating the cytotoxicity of ellipticine in human neuroblastoma cells. Another important result of the study is finding that ellipticine can induce resistance to this agent, which is, however, lower than resistance of neuroblastoma cells to doxorubicin caused by doxorubicin. This finding is the promising result that might be utilized for the development of new neuroblastoma therapies, namely, for the substitution and/or combination of the current anticancer drugs with ellipticine or its derivatives. For the practical use it seems to be important that there is only partial cross-resistance between ellipticine and doxorubicin in neuroblastoma, which is not caused in all cell lines by P-glycoprotein.
